# Green Space, Air Pollution, Weather, and Cognitive Function in Middle and Old Age in China

**DOI:** 10.3389/fpubh.2022.871104

**Published:** 2022-05-02

**Authors:** Lingling Zhang, Ye Luo, Yao Zhang, Xi Pan, Dandan Zhao, Qing Wang

**Affiliations:** ^1^Department of Nursing, University of Massachusetts Boston, Boston, MA, United States; ^2^Department of Sociology, Anthropology and Criminal Justice, Clemson University, Clemson, SC, United States; ^3^Department of Sociology, Texas State University, San Marcos, TX, United States; ^4^Department of Biostatistics, Shandong University, Jinan, China

**Keywords:** green space, air pollution, weather, cognition, middle-aged and older adults

## Abstract

Prior research has shown that environmental hazards, such as limited green space, air pollution, and harmful weather, have the strong adverse impact on older adults' cognitive function; however, most of the studies were conducted in developed countries and limited to cross-sectional analyses. China has the largest aging population in the world so the research evidence from it can offer an insight to the study in other developing countries facing similar issues and inform future public health policy and disease control. This study examined the long-term impact of environmental factors, namely, green space coverage, air pollution, and weather conditions on cognitive function using a nationally representative sample consisting of adults aged 45 years and older selected from the China Health and Retirement Longitudinal Study (CHARLS 2011–2018), the China City Statistical Yearbook, and other sources. Multilevel growth curve models were utilized for analysis and the mediator effects of physical activity and social engagement on the relationship between environmental factors and cognitive function were examined. Findings of this study showed that after controlling for sociodemographic characteristics, annual precipitation of 80 cm or more, living in areas with July temperature of 28°C or higher, urban community, and green space coverage were positively associated with cognition score at the baseline and lower precipitation, urban community, and greater green space coverage were associated with slower cognitive decline over a 7-year period. The impact of gross domestic product (GDP) seemed to take into effect more and more over time. These effects did not substantially change after weekly total hours of physical activities and levels of social engagement were added. More research on the mechanisms of the effect of environmental factors on cognition is needed such as the subgroup analyses and/or with more aspects of environmental measures.

## Introduction

Population aging is a global phenomenon. The world's population aged 60 years and older is estimated to reach 2 billion in 2050 that nearly doubles the increase from 2015, almost with 120 million living in China alone (1). The global population aged 80 and older is expected to grow from 126.5 million in 2015 to 446.6 million in 2050 (1). However, the prolonged life expectancy did not necessarily bring a better quality of life (QoL) (2). On the contrary, it may be coupled with an increased risk of disease, disability, dementia, and advanced aging before death (3), which contribute to the worse QoL among older adults. Therefore, research on health and wellbeing, and how to improve the QoL for the older population is significant and is increasingly important (4, 5).

Cognitive function is essential to QoL and survival in old age (6, 7). Cognitive decline predicts the deterioration of wellbeing, disability, and the frequent use of healthcare; it also affects the activities of daily living of an individual (8). It increased the pressure on the economy, social system, and healthcare (2). Thus, it is urgent to learn the risk factors that contribute to cognitive impairment.

Studies have shown hazard environments such as air pollution, extreme weather, and lack of greenness could affect cognitive function (9). The existing research examining air quality, weather, and green space on other health outcomes, for example, preterm birth (10), led to the focus of environmental factors in this study. A recent meta-review indicated that air pollution exposure had a significant association with cognitive decline, not only mild cognitive impairment but also Alzheimer's disease and dementia (11). The longer the exposure, the more risk for cognitive dysfunction (11). The other two studies also confirmed the adverse effect of air pollution on cognitive function (12, 13). In addition, the review by Power et al. (14) described the negative association between traffic-related air pollution, O_3_ exposure, and dementia-related outcome. However, cross-sectional and longitudinal studies conducted in the United Kingdom showed that the association between air pollution exposure and cognitive performance was weak (15).

Weather is another potential factor relating to cognitive function. A focused review suggested extreme environmental (heat, hypoxia, and cold) conditions affected cognitive function by activity-related stressors (16). However, the data from Schlader et al. (17) study showed that heat stress was unrelated to cognitive function and indicated that the cognitive response to the perceived increase in body temperature was the same among both older and younger adults. A recent study on a large cohort of older Americans found a mixed relationship between the frequency of exposure to precipitation and cognitive function as both positive and negative consequences were achieved on the cognitive function of the older adults depending on the exposure level (18). The data from the study by Dai et al. (19) suggested that the risk of cognitive decline was linked to either high or low temperature in older men.

The residential environment, including green space, has been related to cognitive function as well. A 10-year follow-up cohort study observed that the more green space around the neighborhood the slower cognitive decline during this 10-year follow-up period among middle-aged and older adults (20). Another population-based cohort study indicated that the green space was a protective factor for cognitive decline (21).

The relationship between environment and cognitive function has been identified in existing studies (22, 23). However, most of the studies were cross-sectional and conducted among developed countries and the results were mixed. The data from developing countries are scarce and limited. According to the data from the WHO (1) and the National Institutes of Health (NIH) (24), in 2050, 80% of older people will be living in low- and middle-income countries and the population aged 80 years or older in some Asian and Latin American countries are predicted to quadruple. Thus, this study aimed to examine the long-term impact of environmental factors, namely, green space coverage, air pollution, and weather conditions on cognitive function in China. It will fill the gap in the literature on the environment-cognition relationship in developing countries and provide evidence for future disease control and health policy formulation aiming to slow cognitive decline among middle-aged and older adults.

Furthermore, we acknowledged and examined two subdomains of cognitive function in relation to environmental factors. McArdle et al. (25) study of the Health and Retirement Study data suggested two factors to adequately capture cognitive function: one related to episodic memory and the other related to mental status. Episodic memory is an important aspect of fluid intelligence that concerns learning performance and processing of new materials and tends to decline substantially in adulthood (26). The tasks measuring mental status contain elements of both fluid and crystallized intelligence (26). Crystallized intelligence includes knowledge and skills accumulated in the past, which are not easily lost (26). Although previous research on older Chinese adults have studied these two domains (27, 28), to our knowledge, none has yet examined how they may be differently affected by environmental features.

In addition, both the physical activity participation and social activity participation may mediate the relationship between environmental factors and cognition (23, 29, 30). Physical activities affect cognition positively (31, 32), and so as social activities (28, 33). Improved green space, air quality, and weather could increase physical activity participation (34, 35) and social activity participation (36). However, research on the role of physical and social activity participation in the relationship between environmental factors and cognitive function is limited. In this study, we examine such mediating relationships.

## Methods

This study used data from four waves of China Health and Retirement Longitudinal Study (CHARLS) (2011–2018) and other sources. CHARLS is a longitudinal national survey of middle-aged and older adults in China (37). The CHARLS sample was obtained through multistage stratified probability proportional to size sampling. The baseline survey was conducted in 2011 covering 28 provinces, 150 counties/districts, 450 communities, 10,257 households, and 17,706 respondents aged 45 years and older and their spouses, if available, with an overall response rate of 80.5%. Three follow-up interviews were conducted in 2013, 2015, and 2018. We restricted our analysis to the 17,225 respondents aged 45 years and older at the baseline survey. Among them, 15,547 were reinterviewed in 2013, 14,747 were reinterviewed in 2015 and 14,014 were reinterviewed in 2018. Our analytical sample was further limited to those who were interviewed in 2011, were not missing on basic demographic variables at the baseline survey, and answered cognition questions in at least one wave. The final sample for our analysis included 16,337 respondents in 448 communities, which belong to 125 prefectures. Community-level variables came from CHARLS's Community Survey collected in the baseline survey year 2011. The community-level analyses were conducted at the prefecture level as only the prefecture-level cities or other types of prefecture units are available in the publicly released CHARLS data. To match the data at the same level, we extracted prefecture-level gross domestic product (GDP), population density, and green space coverage from China City Statistical Yearbook and other local yearbooks. For the same reason, environmental variables were also aggregated at the prefecture level. We collected weather data from the Physical Sciences Laboratory of the University of Delaware Air Temperature & Precipitation V5.01 (38) and PM_2.5_ air pollution data from the Atmospheric Composition Analysis Group (39). Although prefecture is a broader and larger unit than community, recent studies have done analyses on prefecture-level environmental factors and older adult's health and wellbeing and found significant associations (40–43). A map of surveyed prefectures and the number of communities in each prefecture in a table are provided in the Supplementary Materials.

### Cognitive Function

Cognitive function data were obtained from CHARLS. CHARLS included the Mini-Mental State Examination (44) which is similar to the US Health and Retirement Study. It assessed memory through an immediate word recall asking respondents to immediately repeat in any order ten Chinese nouns just read to them, and a delayed recall asking respondents to repeat these words 4 min later. It assessed orientation by asking respondents to name the date (month, day, and year), season, and day of the week, assessed visuoconstruction by asking respondents to accurately redraw a previously shown picture, and assessed numeric ability through the serial sevens test which asked respondents to subtract 7 from 100 (up to five times). Following studies using the Health and Retirement Study (45), we constructed a total cognition score ranging from 0 to 31 by summing scores on immediate recall (10 points), delayed recall (10 points), orientation (5 points), visuoconstruction (1 point), and numeric ability (5 points) with a reliability score of 0.71 at the baseline survey. Two subscales were also constructed and analyzed. Episodic memory was measured with the sum of scores on immediate recall and delayed recall (20 points), and mental status was measured with the sum of scores on orientation, visuoconstruction, and numeric ability (11 points).

### Environmental Variables

Based on the data availability, environmental variables in this study were collected at the prefecture level, including green space coverage, air pollution, and weather conditions. Green space coverage was the percentage of built-up areas that were covered by green vegetation, and 2011 green space coverage was extracted from the 2012 City Statistical Yearbooks. PM_2.5_ refers to atmospheric particulate matter (PM) that has a diameter of <2.5 μm and PM_2.5_ level and has been linked to cognitive outcomes (11). China regional PM_2.5_ (V4.CH.03) was estimated by combining Aerosol Optical Depth (AOD) retrievals from the NASA's Medium Resolution Imaging Spectrometer (MODIS), Multiangle Imaging Spectrometer (MISR), and Wide Field Ocean Observation Sensor (SeaWiFS) satellite instruments and coincident aerosol vertical profiles with the GEOS-Chem chemical transport model, and subsequently calibrated to regional ground-based observations using geographically weighted regression (GWR) and it provided annual time series of PM_2.5_ concentration with approximately 1 km × 1 km resolution (46, 47). The weather data from Udel-AirT_Precip V5.01 were mainly drawn from the Global Historical Climatology Network and Legates and Willmott's station records of monthly and annual mean air temperature and total precipitation, which provided monthly time series of surface air temperature and precipitation with approximately 50 km × 50 km resolution (48). The resolution was in line with the existing research by Vavrus and Behnke (49) and Guo et al. (50). Another study indicated very similar simulations between regional climate models (RCMs) at 25 km resolution and 50 km resolution for changes of temperature-related extremes and precipitation-related extremes (51). We applied ArcGIS zonal statistics to extract annual average PM_2.5_, monthly temperatures, and annual total precipitation for each prefecture. We used the average annual PM_2.5_ from 2000 to 2010 to capture the possibly delayed effect of air pollution on cognitive function (52). The temperatures in January and July and annual precipitation were the average for 1981–2010 and were dichotomized to capture extreme weather conditions: annual total precipitation (≥80 cm), temperatures in January (< -10°C/14°F) and July (≥28°C/82.4°F), similar to the use of these variables for weather conditions in the study by Zeng et al. (52).

### Physical and Social Activity Participation

The CHARLS asked about three categories of physical activities: (i) vigorous physical activities that required hard/high-intensity physical effort, (ii) moderate physical activities that required a moderate physical effort, and (iii) walking at home or at work for travel, recreation, or leisure. For each category of physical activities, the respondents were asked whether they performed these activities for at least 10 min continuously in a usual week, the number of days they performed them, and how much time they spent on those days. Based on this information, we calculated the total number of hours a respondent spent on physical activities in a normal week. To obtain a normal distribution, the log form of the variable was calculated and analyzed.

Social activity participation was measured with the count of eight social activities each respondent participated in the month before the interview (interacted with friends; played Ma-Jong, chess, cards, or went to a community club; provided help without pay to family, friends, or neighbors who did not live with the respondent; went to a sporting, social or other kinds of the club; took part in a community-related organization; did voluntary or charity work; cared for a sick or disabled adult who did not live with the respondent without pay; and attended an educational or training course); it ranged from 0 to 8.

### Individual-Level and Community-Level Covariates

The characteristics of the respondents, namely, age in years, gender, education level (no education, primary school, middle school, and above), marital status (married vs. unmarried), living arrangement (living alone or with spouse only, living with children, and living with others), and annual household expenditure, were controlled. The existing literature has shown that expenditures are a better measure of economic resources available to the family than income in developing countries (53). Prefecture-level GDP in Chinese *yuan* (CNY), population density, and whether the respondent lives in an urban community or rural village were also included as control variables. The summed total of annual household expenditures and prefecture-level GDP were log-transformed to correct skewness.

### Statistical Analysis

Descriptive statistics for individual and community characteristics were first calculated. As we acknowledged that environmental factors were correlated with each other, as indicated in the study by Li et al. (54), before estimating the models, we examined correlations among environmental measures and found a high correlation between average monthly temperature and annual precipitation (*r* = 0.77). To avoid underestimation of their effects, we dichotomized temperature and precipitation measures following the use of these variables in the study by Zeng et al. (52). The five environmental measures included in this study had the size of correlations ranging from 0.07 to 0.48. Tolerance scores based on ordinary least squares (OLS) regression using only the baseline data showed that the tolerance scores for these measures ranged from 0.55 to 0.89. Then, to take full advantage of the four waves of data on cognitive function, we used multilevel linear growth curve models to examine the relationship between environmental features on the initial level and trajectory of cognitive function over the 7-year study period. Such models belong to a general class of multilevel models that take into consideration the clustering of observations (within persons and communities) by allowing residuals to be distributed and related across persons and communities (55–57). Four occasions of measurement of cognitive function were nested within individuals who were nested within communities and prefectures. Each respondent's cognitive function scores over the 4 waves were a function of time (time = 0 at baseline survey year 2011 and the value on time indicates years since baseline, ranging from 0 to 7). We specified the intercept(s) (baseline cognitive function scores) and slope(s) (yearly rate of change in cognitive function scores) as random. We used the MIXED procedure in Stata Version 14 to estimate multilevel linear growth curve models using the maximum likelihood method.

Four models were estimated for the overall cognition score first and then separately for episodic memory and mental status. The first model included environmental features and individual-level controls. The second model added measures of community socioeconomic characteristics, namely, urban/rural community, prefecture-level GDP, and population density, to see whether they explain some of the associations between environmental features and cognitive function. The third and fourth models added physical activity participation and social activity participation separately into Model 2 which allowed us to examine whether physical activity participation and social activity participation mediate the relationship between environmental features and cognitive function.

## Results

### Descriptive Statistics

Table 1 shows descriptive statistics. The average score at the baseline was 14.75 for overall cognition, 7.04 for episodic memory, and 7.52 for mental status, and all the three cognitive function measures declined at each follow-up survey. About 51% of the respondents were female. The mean age was 58.83 years. About 27% of respondents did not have any education, 40% had primary school education, and 34% had an education higher than primary school. Over 81% of respondents were married and living with a spouse. About 60% of respondents were living with their children and 31% were living alone or only with their spouse. The average annual household expenditure was CNY 21,747 (about $3,425 with the currency exchange rate as of 6.35). On average, the participants had about 26 h of physical activity in a usual week and less than one social activity participation in the past month. Among the communities included in the study, the average annual PM_2.5_ was 44.34 μg/m^3^ which was higher than the most recent standard of 35 μg/m^3^ published by the China Ministry of Ecology and Environment (58) and much higher than the revised standard of 12 μg/m^3^ published by the US Environmental Protection Agency (EPA) (59). The built area green coverage rate was 37.8%. About 14% of the communities had an average January temperature below −10°C, 22% had an average July temperature of 28°C or higher, and 61% had average annual precipitation of 80 cm or more. One-third of the communities were urban communities. The average prefecture-level GDP in 2011 was CNY 240,665 million (about $37,900 million with the currency exchange rate as of 6.35) and population density is 468 persons/km^2^.

**Table 1:** Descriptive statistics for individual and community characteristics.

**Variables**	**Mean (SD) /Percent**
**Individual characteristics (*N* = 16,337)**	
Cognition 2011^a^	14.75 (5.51)
Cognition 2013	14.13 (6.01)
Cognition 2015	13.47 (6.08)
Cognition 2018	12.37 (7.18)
Episodic memory 2011	7.04 (3.48)
Episodic memory 2013	6.82 (3.69)
Episodic memory 2015	6.18 (3.76)
Episodic memory 2018	5.92 (4.69)
Mental status 2011	7.52 (3.13)
Mental status 2013	7.31 (3.26)
Mental status 2015	7.29 (3.24)
Mental status 2018	6.45 (3.26)
Age	58.83 (9.56)
Female	51.4
**Education**	
None	26.8
Primary school	39.6
Middle school and above	33.6
Married	81.2
**Living arrangement**	
Alone/with spouse only	31.4
With children	59.7
With other relatives	8.9
Annual household expenditure in CNY	21,747 (19,857)
Physical activity hours per week	26.33 (16.81)
Social activities	0.70 (0.85)
**Community characteristics (*N* = 448)**	
Average PM_2.5_ in μg/m^3^ in 2000 to 2010	44.34 (18.53)
Greenspace coverage rate in percentage	37.80 (7.51)
Average January temperature in 1981-2010 < −10°C	14.1
Average July temperature in 1981-2010 ≥ 28°C	22.2
Average annual precipitation in 1981-2010 ≥ 80 cm	60.9
Urban community	33.5
GDP in CNY100 million	2,406 (3.058)
Population density in 1,000 persons/km^2^	0.47 (0.33)

*Unless otherwise specified, these descriptive statistics are for the baseline survey year (2011)*.

a *Number of cases for cognition measures is 13,967 in 2011, 13,356 in 2013, 13,260 in 2015, and 12,046 in 2018*.

### Environmental Features and Baseline Cognitive Function

Table 2 reports the results from multilevel growth curve models for the overall cognition score. Among the environmental features, controlling for individual demographics and other environmental features, green space coverage rate, July temperature, and annual precipitation were significantly associated with the baseline (time = 0) cognitive score (Model 1). The cognitive score would improve by 0.05 points if the built area green coverage rate increased by 1%. Living in areas with annual precipitation of 80 cm or more was associated with 0.58 points lower in the cognitive score. Living in areas with a July temperature of 28°C or higher was associated with 0.57 points higher in the cognitive score. These associations were attenuated when community socioeconomic characteristics were added in Model 2, but green space coverage and annual precipitation remained significant at *p* < 0.01 level while July temperature became marginally significant. Living in an urban community was associated with 1.43 points higher in the cognitive score (*p* < 0.01). When physical activity participation was added in Model 3 and social activity was added in Model 4, both the physical activity participation and social activity participation were positively associated with baseline cognitive function. However, there was little change in the associations between environmental features and baseline cognitive score. The annual PM_2.5_ averaged from 2000 to 2010 was not significantly associated with baseline cognitive function.

**Table 2:** Regression coefficients from multilevel linear growth curve model of trajectories of cognitive function in mid and later life.

**Variables**	**Model 1**	**Model 2**	**Model 3**	**Model 4**
Change rate: Time	−0.54^**^	−0.79^**^	−0.80^**^	−0.79^**^
Intercept	9.37^**^	9.58^**^	9.25^**^	9.54^**^
Individual level controls				
Female	−0.50^**^	−0.54^**^	−0.50^**^	−0.52^**^
Age (centered at 45)	−0.13^**^	−0.13^**^	−0.13^**^	−0.13^**^
Education (ref = none)				
Primary school	4.34^**^	4.29^**^	4.29^**^	4.25^**^
Middle school and above	6.95^**^	6.81^**^	6.83^**^	6.68^**^
Married	0.47^**^	0.47^**^	0.46^**^	0.53^**^
Living arrangement (ref = alone or with spouse only)				
Living with children	−0.31^**^	−0.30^**^	−0.30^**^	−0.26^**^
Living with others	0.01	0.02	0.01	0.03
Annual household expenditure (log)	0.20^**^	0.19^**^	0.19^**^	0.17^**^
**Community characteristics**				
PM_2.5_ in μg/m^3^	−0.004	−0.01	−0.01	−0.01
Greenspace coverage rate in percentage	0.05^**^	0.03^**^	0.03^**^	0.03^**^
January temperature < −10 °C	0.38	0.21	0.19	0.17
July temperature ≥ 28 °C	0.57^**^	0.33	0.34	0.34^*^
Annual precipitation ≥ 80 cm	−0.58^**^	−0.45^**^	−0.47^**^	−0.47^**^
Urban community		1.43^**^	1.50^**^	1.33^**^
GDP in CNY100 million (log)		0.02	0.02	−0.01
Population density in 1,000 persons/km^2^		0.41	0.41	0.42
**Mediators**				
Physical activity hours per week (log)			0.10^**^	
Social activities				0.59^**^
**Change rate by community characteristics**				
Time ^*^ PM_2.5_ in ug/m^3^	0.001	0.001	0.001	0.001
Time ^*^ Greenspace coverage rate in percentage	0.01^**^	0.002	0.002	0.002
Time ^*^ January temperature < −10 °C	0.08	0.05	0.05	0.05
Time ^*^ July temperature ≥28 °C	−0.04	−0.06	−0.06	−0.06
Time ^*^ Annual precipitation ≥80cm	−0.15^**^	−0.13^**^	−0.13^**^	−0.13^**^
Time ^*^ Urban community		0.14^**^	0.14^**^	0.14^**^
Time ^*^ GDP in CNY100 million (log)		0.05^**^	0.05^**^	0.05^**^
Time ^*^ Population density in 1,000 persons/km^2^		−0.06	−0.07	−0.07
**Change rate by mediators**				
Time ^*^ Physical activity hours per week (log)			0.002	
Time ^*^ Social activities				0.003
**Random effects**				
Community level variance				
Change rate	0.05^**^	0.04^**^	0.04^**^	0.04^**^
Intercept	1.76^**^	1.27^**^	1.29^**^	1.15^**^
**Individual level variance**				
Change rate	0.16^**^	0.16^**^	0.16^**^	0.17^**^
Intercept	8.51^**^	8.50^**^	8.47^**^	8.29^**^
Residual variance	11.17^**^	11.18^**^	11.17^**^	11.17^**^
Observations	52,629	52,629	52,629	52,629
Number of groups	448	448	448	448
Chi-square	13,350	13,827	13,876	14,348
df	19	25	27	27

** *p < 0.01*,

* *p < 0.05 (two-tailed tests)*.

Table 3 reports the results from multilevel growth curve models for episodic memory and mental status. Controlling for individual demographics and other environmental features, PM_2.5_ and high annual precipitation were negatively associated with baseline episodic memory score (“Episodic memory”: Model 1) while high July temperature was positively associated with baseline episodic memory score. Annual precipitation remained significant after community socioeconomic characteristics were added in Model 2. There was not much change in these associations after physical activity and social activity measures were added in Model 3 and Model 4. Urban community, physical activity, and social activity were positively associated with baseline episodic memory. Green space coverage range, low January temperature, and high July temperature were positively associated with mental status (“Mental status”: Model 1). These associations were attenuated after community socioeconomic characteristics were added in Model 2, but green space coverage rate and January temperature remained significant. There was not much change in these associations after physical activity and social activity measures were added in Model 3 and Model 4. Urban community, GDP, population density, physical activity, and social activity were positively associated with mental status.

**Table 3:** Regression coefficients from multilevel linear growth curve model of trajectories of episodic memory and mental status in mid and later life.

	**Episodic memory**				**Mental status**			
**Variables**	**Model 1**	**Model 2**	**Model 3**	**Model 4**	**Model 1**	**Model 2**	**Model 3**	**Model 4**
Change rate: Time	−0.40^**^	−0.59^**^	−0.60^**^	−0.59^**^	−0.12^**^	−0.18^**^	−0.17^**^	−0.18^**^
Intercept	5.61^**^	6.35^**^	6.18^**^	6.33^**^	3.48^**^	2.94^**^	2.73^**^	2.92^**^
**Community characteristics**								
PM_2.5_ in μg/m^3^	−0.01^*^	−0.01	−0.01	−0.01^*^	0.004	0.0003	0.0002	−0.0003
Greenspace coverage rate in percentage	0.01	0.004	0.004	0.004	0.04^**^	0.02^**^	0.02^**^	0.02^**^
January temperature < −10 °C	0.04	−0.07	−0.07	−0.09	0.37^**^	0.31^*^	0.30^*^	0.29^*^
July temperature ≥28 °C	0.25^*^	0.19	0.19	0.19+	0.31^**^	0.13	0.13	0.13
Annual precipitation ≥80cm	−0.42^**^	−0.39^**^	−0.40^**^	−0.40^**^	−0.15	−0.05	−0.06	−0.06
Urban community		0.63^**^	0.66^**^	0.57^**^		0.79^**^	0.84^**^	0.75^**^
GDP in CNY100 million (log)		−0.11	−0.11	−0.13^*^		0.13^**^	0.13^**^	0.12^**^
Population density in 1,000 persons/km^2^		0.09	0.09	0.09		0.41^**^	0.41^**^	0.41^**^
**Mediators**								
Physical activity hours per week (log)			0.05^**^				0.07^**^	
Social activities				0.34^**^				0.27^**^
**Change rate by community characteristics**								
Time ^*^ PM_2.5_ in ug/m^3^	0.001	0.001	0.001	0.001	−0.0004	0.0001	0.0001	0.0001
Time ^*^ Greenspace coverage rate in percentage	0.01^**^	0.003^*^	0.003^*^	0.003^*^	−0.0001	−0.001	−0.001	−0.001
Time ^*^ January temperature < −10 °C	0.08^*^	0.06	0.06	0.06	−0.01	−0.01	−0.01	−0.01
Time ^*^ July temperature ≥28 °C	−0.03	−0.05^*^	−0.05^*^	−0.05^*^	−0.005	0.0003	0.0002	0.0005
Time ^*^ Annual precipitation ≥80cm	−0.13^**^	−0.11^**^	−0.11^**^	−0.11^**^	−0.02	−0.02	−0.02	−0.02
Time ^*^ Urban community		0.12^**^	0.12^**^	0.12^**^		0.02	0.02	0.02
Time ^*^ GDP in CNY100 million (log)		0.04^**^	0.04^**^	0.04^**^		0.01	0.01	0.01
Time ^*^ Population density in 1,000 persons/km^2^		−0.01	−0.01	−0.01		−0.07^*^	−0.07^**^	−0.07^**^
**Change rate by mediators**								
Time ^*^ Physical activity hours per week (log)			0.004				−0.005^*^	
Time ^*^ Social activities				0.002				−0.004

*Individual level covariates were included in all models (details available upon request)*.

** *p < 0.01*,

* *p < 0.05 (two-tailed tests)*.

### Environmental Features and Cognitive Function Trajectories

In Table 2, green space coverage rate and annual precipitation were significantly associated with the overall cognitive function trajectories over the 7-year study period (Model 1). 1% increase in green coverage rate lowered the decline rate in the overall cognitive score by 0.01 points, and annual precipitation of 80 cm or more was associated with 0.15 points faster decline rate in the overall cognitive score. When community socioeconomic characteristics were added in Model 2, green space coverage rate was no longer significantly associated with the change in cognitive score, but annual precipitation remained significant. Urban community and one-point higher in prefecture-level GDP (log) lowered cognitive decline rate by 0.14 and 0.05 points, respectively. There was no significant association between the annual PM_2.5_ averaged from 2000 to 2010 and the cognitive decline rate. When physical activity participation was added in Model 3, physical activity participation was not significantly associated with the rate of decline in the cognitive score and there was little change in the association between environmental features and cognitive decline rate. When social activity participation was added in Model 4, social activity participation was not significantly associated with cognitive decline rate and there was little change in the associations between environmental features and cognitive decline rate. Among other individual-level covariates, the overall cognitive scores among women were 0.5–0.54 points lower than men (*p* < 0.01). As age increased 1 year, the overall cognitive score declined 0.13 points across the models (*p* < 0.01). In comparison with people without any education, the cognitive scores of those with primary education and middle school or more education were 4.25–4.34 points higher and 6.68–6.95 points higher, respectively (*p* < 0.01). The respondents who were married and living with their spouse had 0.46–0.51 points higher cognitive scores than those unmarried respondents (*p* < 0.01). The respondents living with children were 0.28–0.31 points lower in baseline cognitive score across models (*p* < 0.01). When the annual household expenditure (log) increased by one unit, the cognitive score increased 0.18–0.2 points (*p* < 0.01).

In Table 3, green space coverage rate, January temperature, and annual precipitation were significantly associated with the trajectories of episodic memory over the 7-year study period (“Episodic memory”: Model 1). While higher green space coverage and lower January temperature lowered the decline rate in episodic memory, higher precipitation was associated with a faster decline in episodic memory. Green space coverage rate and precipitation remained significant after community socioeconomic characteristics were added in Model 2 and these associations did not change much when physical activity and social activity measures were added in Model 3 and Model 4. Urban community and higher GDP were associated with a slower decline rate in episodic memory. Except for population density being associated with faster decline in mental status, none of the environmental features were significantly associated with the rate of decline in mental status, which could be attributable to crystallized intelligence of mental status not easily lost as fluid intelligence of episodic memory over time thus might be less responsive to environmental factors. The associations between individual-level covariates and the two subdomains of cognitive function were generally consistent with the patterns found in overall cognition with one exception that women were 0.25 points higher than men in episodic memory but 0.73 points lower than men in mental status.

## Discussion

In this study, we examined the relationship between environmental features and cognitive trajectories among middle-aged and older adults in China. After controlling for sociodemographic characteristics, we found the positive associations of environmental factors with baseline cognitive function from the higher rate of built area green coverage on overall cognition and mental status, January temperature below −10°C on mental status, and July temperature of 28°C or higher on all the cognitive measures, while higher precipitation seemed to be detrimental for overall cognition and episodic memory. PM_2.5_ had a negative impact on episodic memory only. These findings are consistent with existing literature (18–20, 60). Green space would be beneficial for both physical and mental health, as previous studies showed (35). People tend to exercise more and/or experience a better mood when there is more green coverage. The impact of precipitation might be due to the effect on mobility (36) and decreased exposure to sunlight (61). With higher precipitation, people might be less likely to go outside for physical and/or social activities (34, 62). We also found positive associations between urban community and GDP and cognitive function which are consistent with the literature. Being in an urban community or a more affluent or populous region was likely to slow down cognitive decline as urban communities may provide more resources such as public facilities, healthcare, easy access to local services, and so on (63–65).

The growing evidence suggests that air pollution, especially PM, is associated with cognitive decline (12, 66). Our results were mixed on the air pollution effect. Based on the available data, we found a negative association between PM_2.5_ and episodic memory, but PM_2.5_ was not significantly associated with the overall cognition score and mental status in our data. One explanation may be as Zeng et al. (52) pointed out in their study that the non-statistically significant association between air pollution and mortality was possible because the follow-up period was not long enough. The same reason could be applied to the relationship between air pollution and cognition in our finding. The other attributing factor might be the variations in estimating cognitive function. There are several other studies showing less consistent results for various reasons. In Kulick et al. (67) study, the authors achieved mixed results by revealing the significant association between residential air pollution and cognitive function and decline among older adults in one cohort but not the other in the northern Manhattan area of New York City. A study by Gatto et al. (68) found no association between air pollutants and a global cognitive score among middle-aged and older adults in Los Angeles. The study on the relationship between PM_2.5_ and incident cognitive impairment by Loop et al. (69) stated that the evidence was lacking to achieve a robust association. Given the mixed results, the conclusion on the findings of this study should be drawn with caution.

If taking into consideration of cognitive trajectories, the environmental factors of annual precipitation of 80 cm or more, urban community, and green space coverage at the baseline remained significant and lower precipitation, urban community, and greater green space coverage are associated with slower overall cognitive decline over a 7-year period. In addition, the impact of GDP (log) became statistically significant. It seems that in a long run, the influence from economic development may take into effect more and more over time. The results of episodic memory shared a similar trend as overall cognition but green space coverage had a more robust effect on episodic memory across models. The over-time higher July temperature was associated with a faster decline in episodic memory after controlling community socioeconomic characteristics. The temperature might affect cognition through physical, mental, or physiologic impact, but the influencing factors were not exhaustive in this study. The advantage of living in a more urbanized community diminished over time for mental status and the higher population density could even bring adverse effects. As explained in the study by Xiang et al. (65), high population density is one downside among many that are coupled with China's drastic rise in urbanization in the recent decades. They all lead to more and more constricted life space for the senior population, which has been shown to be associated with cognitive decline (65).

In accordance with previous literature, physical activity participation and social activity participation were positively associated with all cognitive measures among middle-aged and older Chinese adults in this analysis. However, the relationship between environmental factors and cognitive function did not substantially change after weekly total hours of physical activities and levels of social engagement were added, and thus the physical and social activity participation did not seem to mediate the relationship between environmental factors and cognitive function. The finding suggested that Chinese middle-aged and older adults do not necessarily reduce their physical and social activities when facing unfavorable environmental conditions. A caution was raised for mental status as our results showed that the positive association between physical activity at baseline and cognitive function might level off as time passed by. This seemed to suggest physical activity participation should be maintained over time to fully benefit from it. More research is needed to identify other possible mechanisms in the relationship between environmental factors and cognitive function.

### Limitation

Due to the data availability, the measures for environmental factors were not ideal or comprehensive enough. The data on green space, air pollution, and weather conditions were extracted and merged from different sources which were constrained by data source limitation and accuracy. For instance, the analyses could only be done at the prefecture level for selected years. The intense rainfall might be underestimated given that it could be highly localized but was spatially averaged in the RCMs over 50 km × 50 km resolution (49). Furthermore, the statistical analyses indicated an association between prior environmental factors and cognitive function and cognitive change over time, but it did not track changes in environmental factors over time. Future study is warranted to examine the full impact of the environment on cognition and establish causal pathways. Despite these limitations, this study performed a rigorous test using longitudinal data from a national representative sample of older adults in China and combined the data of environmental features to provide a new perspective on the environmental influence on cognitive trajectories among middle-aged and older adults. It led the way for potential exploration and more in-depth analyses in this realm.

## Conclusion

This study examined the effect of environment on cognitive function. The results added value to the existing knowledge of the association between environmental factors and cognition. In general, the findings suggested increasing green space coverage which is a readily modifiable factor, improving socioeconomic conditions, and promoting the level of physical and social activity participation for middle-aged and older adults. Efforts should also be made to mitigate the possible detrimental effect of heavy rainfall on older adult's health and wellbeing and air pollution on cognition. From the policy perspective, a favorable environment for cognitive health would be more beneficial for promoting aging in place.

## Data Availability Statement

The original contributions presented in the study are included in the article/Supplementary Materials, further inquiries can be directed to the corresponding author.

## Author Contributions

LZ and YL contributed to the study conceptualization, data collection, analysis and result interpretation, and manuscript writing. YZ contributed to the literature search and summary. XP, QW, and DZ contributed to the data collection and interpretation. All co-authors reviewed the manuscript and provided their inputs. All authors contributed to the article and approved the submitted version.

## Funding

This work was funded by National Institutes of Health (R03AG065637).

## Conflict of Interest

The authors declare that the research was conducted in the absence of any commercial or financial relationships that could be construed as a potential conflict of interest.

## Publisher's Note

All claims expressed in this article are solely those of the authors and do not necessarily represent those of their affiliated organizations, or those of the publisher, the editors and the reviewers. Any product that may be evaluated in this article, or claim that may be made by its manufacturer, is not guaranteed or endorsed by the publisher.
