# Supplementary material for: Green Space, Air Pollution, Weather, and Cognitive Function in Middle and Old Age in China
Source: Front Public Health. 2022 May 2;10:871104. doi: 10.3389/fpubh.2022.871104 (PMC9108722; doi:10.3389/fpubh.2022.871104)
Supplement: Supplementary file 1 [file Table_1.docx]

**Supplement 1. Number of communities included in the study based on CHARLS 2011**

| Province | Prefecture | Number of communities |
| --- | --- | --- |
| Shanghai | Shanghai | 3 |
| Yunnan | Lincang | 3 |
|  | Lijiang | 3 |
|  | Baoshan | 3 |
|  | Kunming | 6 |
|  | Zhaotong | 6 |
|  | Chuxiong Yi | 6 |
| Inner Mongolia | Xing'an | 3 |
|  | Hulunbuir | 6 |
|  | Hohhot | 3 |
|  | Chifeng | 3 |
|  | Xili Gol | 3 |
| Beijing | Beijing | 3 |
| Jilin | Jilin | 3 |
|  | Siping | 6 |
| Sichuan | Neijiang | 3 |
|  | Liangshan Yi | 3 |
|  | Nanchong | 6 |
|  | Yibin | 6 |
|  | Guang'an | 3 |
|  | Chengdu | 3 |
|  | Garzê Tibetan | 3 |
|  | Meishan | 3 |
|  | Mianyang | 3 |
|  | Ziyang | 3 |
| Tianjin | Tianjin | 3 |
| Anhui | Bozhou | 3 |
|  | Lu'an | 3 |
|  | Anqing | 3 |
|  | Suzhou | 3 |
|  | Chaohu | 3 |
|  | Huainan | 3 |
|  | Fuyang | 3 |
| Shandong | Linyi | 6 |
|  | Weihai | 3 |
|  | Dezhou | 1 |
|  | Zaozhuang | 3 |
|  | Jinan | 6 |
|  | Binzhou | 3 |
|  | Weifang | 3 |
|  | Liaocheng | 3 |
|  | Qingdao | 3 |
| Shanxi | Linfen | 3 |
|  | Xinzhou | 3 |
|  | Yuncheng | 3 |
|  | Yangquan | 3 |
| Guangdong | Foshan | 3 |
|  | Guangzhou | 6 |
|  | Jiangmen | 6 |
|  | Shenzhen | 3 |
|  | Qingyuan | 3 |
|  | Chaozhou | 3 |
|  | Maoming | 9 |
| Guangxi | Nanning | 3 |
|  | Guilin | 3 |
|  | Hechi | 3 |
|  | Yulin | 6 |
| Xinjiang Uygur | Aksu | 3 |
| Jiangsu | Suqian | 3 |
|  | Xuzhou | 3 |
|  | Yangzhou | 3 |
|  | Taizhou | 3 |
|  | Yancheng | 3 |
|  | Suzhou | 3 |
|  | Lianyungang | 3 |
| Jiangxi | Shangrao | 3 |
|  | Jiujiang | 3 |
|  | Nanchang | 9 |
|  | Ji'an | 3 |
|  | Yichun | 3 |
|  | Jingdezhen | 3 |
|  | Ganzhou | 3 |
| Hebei | Baoding | 6 |
|  | Chengde | 3 |
|  | Cangzhou | 3 |
|  | Shijiazhuang | 6 |
| Henan | Xinyang | 6 |
|  | Zhoukou | 3 |
|  | Anyang | 3 |
|  | Pingdingshan | 3 |
|  | Luoyang | 6 |
|  | Puyang | 3 |
|  | Jiaozuo | 3 |
|  | Zhengzhou | 6 |
| Zhejiang | Lishui | 3 |
|  | Taizhou | 6 |
|  | Jiaxing | 3 |
|  | Ningbo | 3 |
|  | Hangzhou | 3 |
|  | Huzhou | 3 |
| Hubei | Enshi Tujia and Miao | 3 |
|  | Jingmen | 3 |
|  | Xiangfan | 3 |
|  | Huanggang | 3 |
| Hunan | Loudi | 3 |
|  | Yueyang | 3 |
|  | Changde | 3 |
|  | Yiyang | 3 |
|  | Shaoyang | 6 |
|  | Changsha | 3 |
| Gansu | Lanzhou | 3 |
|  | Dingxi | 3 |
|  | Pingliang | 3 |
|  | Zhangye | 3 |
| Fujian | Ningde | 3 |
|  | Zhangzhou | 3 |
|  | Fuzhou | 3 |
|  | Putian | 3 |
| Guizhou | Qiandongnan Miao and Dong | 3 |
|  | Qiannan Buyei and Miao | 3 |
| Liaoning | Dalian | 3 |
|  | Chaoyang | 3 |
|  | Benxi | 3 |
|  | Jinzhou | 3 |
|  | Anshan | 3 |
| Chongqing | Chongqing | 6 |
| Shaanxi | Baoji | 6 |
|  | Yulin | 3 |
|  | Hanzhong | 3 |
|  | Weinan | 3 |
| Qinghai | Haidong | 3 |
| Heilongjiang | Jiamusi | 3 |
|  | Harbin | 3 |
|  | Jixi | 3 |
|  | Qiqihar | 3 |
| Total |  | 448 |
