# Supplementary material for: Green Space, Air Pollution, Weather, and Cognitive Function in Middle and Old Age in China
Source: Front Public Health. 2022 May 2;10:871104. doi: 10.3389/fpubh.2022.871104 (PMC9108722; doi:10.3389/fpubh.2022.871104)
Supplement: Supplementary file 2 [file Image_1.pdf]

Supplement 2. Survey map by CHARLS

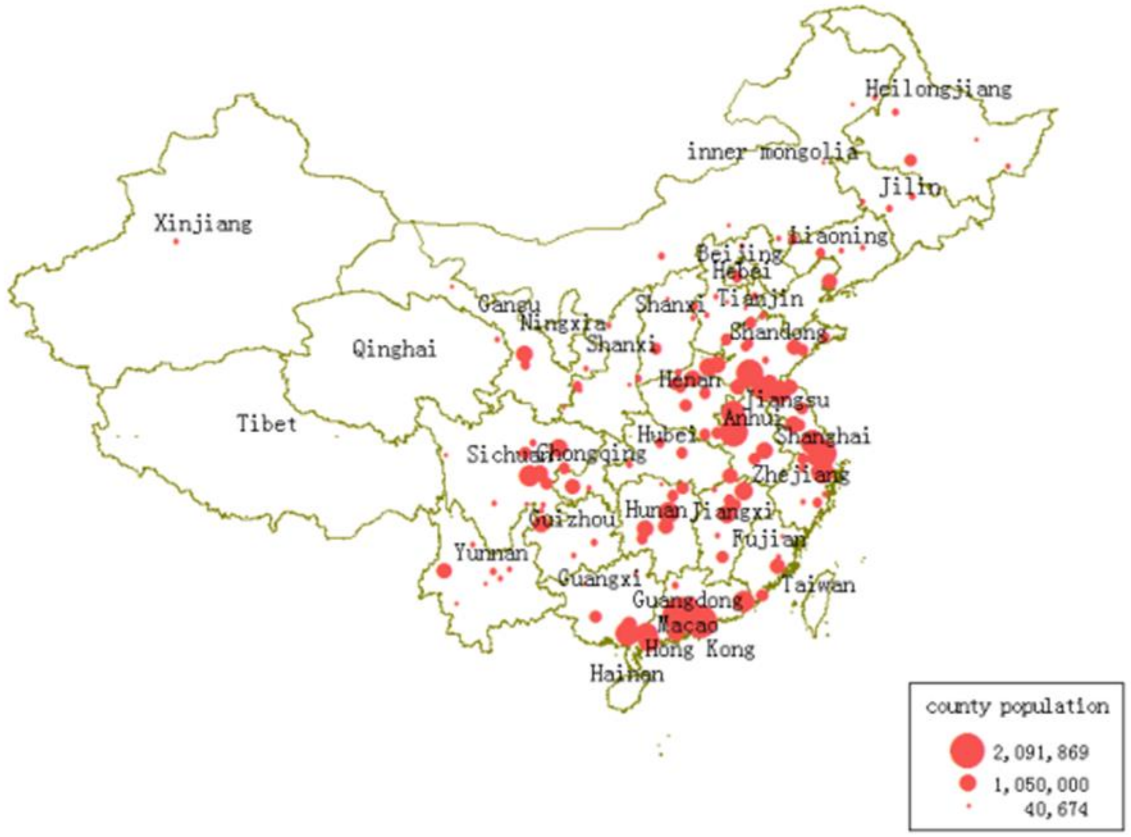

Source: Zhao et al., 2013

REFERENCE

Zhao Y, Strauss J, Yang G, Giles J, Hu P, Hu Y, et al. China Health and Retirement Longitudinal Study – 2011-2012 National Baseline User's Guide. China Health and Retirement Longitudinal Study, Peking University (2013). Available online at: http://charls.pku.edu.cn/en/doc/User2011.pdf (accessed February 2 2, 2022).
